# Prevalence and Characteristics of Social Withdrawal Tendency Among 3–24 Months in China: A Pilot Study

**DOI:** 10.3389/fpsyt.2021.537411

**Published:** 2021-06-16

**Authors:** Fengjuan Zhou, Peiyuan Huang, Xueling Wei, Yixin Guo, Jinhua Lu, Lanlan Feng, Minshan Lu, Xian Liu, Si Tu, Alexandra Deprez, Antoine Guedeney, Songying Shen, Xiu Qiu

**Affiliations:** ^1^Division of Birth Cohort Study, Guangzhou Women and Children's Medical Center, Guangzhou Medical University, Guangzhou, China; ^2^Department of Woman and Child Health Care, Guangzhou Women and Children's Medical Center, Guangzhou Medical University, Guangzhou, China; ^3^Laboratory of Psychopathology and Health Processes, Institute of Psychology, University of Paris Descartes, Paris, France; ^4^Department of Child and Adolescent Psychiatry, Bichat Claude Bernard Hospital, Paris University, Paris, France; ^5^Department of Obstetrics and Gynecology, Guangzhou Women and Children Medical Center, Guangzhou Medical University, Guangzhou, China

**Keywords:** social withdrawal, temperament, scale, anxiety, infant

## Abstract

**Background:** Sustained withdrawal behavior is an obstacle for child development. The present study aimed to preliminarily evaluate the prevalence of social withdrawal tendency in young Chinese children using the Alarm Distress Baby Scale (ADBB) and describe the characteristics of socially withdrawn children.

**Method:** This was a cross-sectional analysis as part of a prospective cohort study. A total of 114 children aged 3–24 months were included. The following instruments were administered: the Chinese version of ADBB, the Ages and Stages Questionnaire (ASQ-3), the Ages and Stages Questionnaire: Social-Emotional (ASQ:SE), and the Infant Temperamental Questionnaire. The tendency of social withdrawal in children was assessed using the ADBB. Social withdrawal was defined as an ADBB score of 5 or above. Student's *t*-test, χ^2^ test, and Fisher's exact test were performed to identify the differences in maternal and child characteristics between the children with and without social withdrawal. Age-specific indicators of development in these two groups were also presented.

**Results:** About 16.7% of the children were socially withdrawn. Compared with those without social withdrawal, children with social withdrawal were older and had higher proportions of boys (68.4 vs. 42.1%) and social-emotional development delay (63.2 vs. 0%). In age-specific analyses, social-emotional development was poorer in children with social withdrawal across all age groups from 3 to 24 months.

**Conclusion:** Assessed by the ADBB, the prevalence of social withdrawal tendency in young Chinese children was similar to that reported in the European population; children with social withdrawal tended to have poorer social-emotional development. Further research with larger sample sizes is needed to validate the scale and confirm these findings.

## Introduction

The social withdrawal behavior of young children is characterized by less positive behaviors, such as avoiding eye contact, smiling, cooing, or negative behaviors, such as self-stimulation (1). Short-time social withdrawal behavior is a normal feature of children's behavior in parent-child interaction, as it provides a way for children to regulate the interaction. However, persistent social withdrawal is considered a defensive mechanism observed when a child is faced with a lack of synchronization within the parent-infant interaction or a lack of repair process from mismatches (2).

The Alarm Distress Baby scale (ADBB) was developed in 2001 by Guedeney et al. and is widely used as the screening tool for social withdrawal in children aged 2–24 months (1). Children with an ADBB score of 5 or above are considered socially withdrawn, accounting for around 13–20% in European countries (3, 4). Evidence has shown that social withdrawal in infancy is associated with a range of developmental difficulties in later life. Children with social withdrawal behavior have a higher risk of late talking in a French birth cohort study (4). Another study also shows that social withdrawal at 12 months of age can predict behavioral, emotional, and social difficulties at late preschool age (5). To investigate the relationship between infant social withdrawal and later developmental outcomes, such as intelligence quotient (IQ), Guedeney et al. conducted a study based on the EDEN mother-child cohort. Social withdrawal was assessed by trained nurses using the ADBB at the age of 1 year; IQ was tested using Wechsler Preschool and Primary Scale of Intelligence – III at 5–6 years of age. Compared to children with an ADBB score <5, those with a score ≥ 5 at age 1 year had significantly lower IQ at age 5–6 years (6). Sustained social withdrawal behavior may be linked with parent-infant relationship disorders, as seen in caregivers with depressive and anxiety disorders, or with processing difficulties of the child (e.g., mental retardation of genetic or perinatal causes, autism, and regulatory disorders) (7, 8).

The Chinese version of the Child Social Preference Scale is used for assessing the shyness and socio-emotional functioning of preschool children at age 3–5 years in China (9). The Ages & Stages Questionnaires: Social-Emotional (ASQ:SE), adapted into China in 2017 by Bian et al. (10), are caregiver-completed questionnaires that evaluate the development of children's socio-emotional functioning. Studies of children in urban China have also found that the withdrawn temperament of young children is related to the increase of internalizing problems in early childhood and externalizing problems in mid- and late childhood (11). However, no studies have evaluated the developmental characteristics of social withdrawal, as measured by the ADBB, in young Chinese children. Therefore, this pilot study aimed to preliminarily evaluate the prevalence of social withdrawal in young Chinese children using the ADBB and describe the characteristics of socially withdrawn children.

## Materials and Methods

### Sample Recruitment and Study Design

This pilot study, conducted between June 2017 and December 2018, was part of the Born in Guangzhou Cohort Study (BIGCS), which is an ongoing prospective birth cohort study conducted by the Guangzhou Women and Children's Medical Center (GWCMC), China (12). The inclusion criteria were: (1) the family was residents in Guangzhou; (2) the child was aged 3–24 months old; (3) the family planned to return to the hospital for follow-ups; (4) the family agreed on having the examination of the child on a video during the follow-up. The exclusion criteria were: (1) multiple gestations, (2) children with obvious motor developmental disabilities, and (3) refusal to collect video data or participate in follow-ups. A total of 120 families were approached, and 114 (95%) completed the follow-up. The study was approved by the Institutional Ethics Committee of the GWCMC (approval number 2015072718). Written informed consent was obtained from each participating family. The registration number of the BIGCS is NCT02526901.

### Procedure

Before the commencement of this study, we contacted the team that developed the ADBB and received their instructions and training. Children were independently assessed using ADBB by two psychological evaluators working at the outpatient clinic of the GWCMC during the routine follow-up of the BIGCS. The total score was computed immediately after each examination was completed. The whole examination procedure took about 20–40 min. At the same visit, parents were also asked to complete a standard Chinese version of the Ages & Stages Questionnaires – Third Edition (ASQ-3) and the Ages & Stages Questionnaires: Social-Emotional (ASQ:SE). Some examinations were recorded by video, keeping attention both on the children's behavior and their reactions to the observers during the examination. Quality control was performed throughout the course of the study. The supervision team, consisting of the first author, Director of the BIGCS, and Head of the BIGCS Child Follow-up Team, conducted on-site inspections on the assessment and data collection process on a monthly basis, performing live examinations for each evaluator, monitoring any assessment difficulties, and reviewing all the data collected.

### Translation and Back-Translation

The translation and adaptation of the Chinese version of ADBB from the English version were conducted according to the instructions provided by Guedeney (1). During this process, a forward translation was processed from the original English version into Mandarin. The first draft was presented to a panel consisting of two neuropsychological nurses and five pediatricians for review, and the translation was amended based on their clinical experience to achieve consensus. The Mandarin version was then back-translated into English by two translators (a bilingual university faculty member with a Master's degree in medical sciences and a bilingual translator with a Master's degree in pedagogy), working independently from one another. By comparing the translated English version with the original English version, the meanings of these entries were found identical.

### Measurement

#### ADBB

In 2001, the ADBB was developed by Guedeney and colleagues for children aged 2–24 months. Consistent with its original version, the Chinese version of ADBB consists of 8 items related to social behaviors and features of the child: facial expression, eye contact, the overall level of activity, self-stimulating gestures, vocalization, briskness of response to stimulation, ability to engage in a relationship, and the attraction between the child and the evaluator. The eight-item scale is measured on a five points scale (0 = definite normal behavior, 1 = discreet social withdrawal behavior, 2 = clear social withdrawal behavior, 3 = obvious social withdrawal behavior, 4 = definite social withdrawal behavior). The total score, ranging from 0 to 32, is the arithmetic sum of all item scores, with a higher score representing a stronger tendency of social withdrawal. The cut-off of 5 has been proven as an acceptable level of sensitivity and specificity for defining social withdrawal in other studies conducted in several countries (1, 13, 14).

#### ASQ-3

The ASQ-3 evaluates the following five developmental domains: gross motor, fine motor, communication, problem-solving (i.e., cognitive), and personal-social, applicable to children aged from 1 month to 5.5 years. Based on the developmental milestones of children at different ages, it has age-specific versions that cover the age intervals of our participants, e.g., the versions for 3 months 0 days−4 months 30 days, 5 months 0 days−6 months 30 days, 11 months 0 days−12 months 30 days, and 23 months 0 days−25 months 15 days, etc. In our survey, the ASQ-3 was administered by trained psychological evaluators who interviewed the primary caregiver of each child. The tool has generally high internal consistency, test-retest reliability, and acceptable sensitivity (15). For more information about the ASQ-3, please refer to: https://agesandstages.com/.

#### ASQ:SE

The ASQ:SE is specifically tailored to assess the social-emotional functioning of children aged from 1 month to 6 years (16). Similar to the ASQ-3, the ASQ:SE also has age-specific versions suitable for the age groups of our participants. In our survey, caregivers were provided with a list of behaviors and required to choose one of the following answers based on the frequency of each behavior in their children: “most of the time,” “sometimes,” or “seldom or never.” Each choice represents a different score for an item, and the ASQ:SE total score is the sum of the score of each item. Unlike the scoring system of the ASQ-3, a lower ASQ:SE score represents better social-emotional development (10). More information about the ASQ:SE can be found at: https://agesandstages.com/.

### Child Temperament

The Chinese version of the Infant Temperamental Questionnaire is a screening tool for child difficulty and assesses the degree to which a child is fussy, unadaptable, unpredictable, and unenjoyable. Mothers were asked to rate their children's behavior using 96 items scored on a 6-point scale from 1 (never) to 6 (almost). The items belong to activity level, rhythmicity, approach/withdrawal, adaptability, intensity of reaction, threshold of responsiveness, quality of mood, distractibility, and attention span and persistence (17).

### Other Variables

Information including maternal socio-demographic data, personal lifestyle, medical histories, psychological status in pregnancy, and birth outcomes was collected by questionnaires or abstracted from medical records at the GWCMC. Maternal mental health was assessed by the Self-rating Anxiety Scale (SAS) and the Self-rating Depression Scale (SDS) between 28 and 40 weeks of gestation. The SAS is a well-studied 20-item scale to measure state and trait anxiety with a 4-point Likert scale in each question; similarly, the SDS is a well-validated scale with 20 items measuring common depressive symptoms (18, 19). Birth weight z-scores were calculated based on the INTERGROWTH-21st standards (20).

### Statistical Analysis

Mean [standard deviation (SD)] and frequency and percentage were used to describe continuous and categorical variables, respectively. Maternal and child characteristics, included maternal age, maternal education level (middle school or below, vocational or technical college, undergraduate, or postgraduate), monthly income ( ≤ 1,500, 1,501–4,500, 4,501–9,000, or ≥9,001 yuan), maternal mental health status (including anxiety and depression), delivery mode, gestational age at delivery, child age, gender, birth order, birth weight z-scores, temperament, and development.

Associations between maternal and child characteristics and the risk of social withdrawal in children were tested using the Student's *t*-test (for continuous variables) or the χ^2^ test (for categorical variables). Fisher's exact test was used instead of the χ^2^ test when any expected frequency was <1 or when ≥20% of the expected frequencies were ≤ 5. Age-specific indicators of development in the children with and without social withdrawal were also presented. A two-tailed *P*-value below 0.05 was considered statistically significant.

## Results

### Maternal and Child Characteristics in Children With an ADBB Score ≥ 5 vs. <5

A total of 114 children aged 3–24 months participated in this pilot study. Table 1 shows the characteristics of the mothers and the children. The mean maternal age at conception was 30.2 (SD 4.0) years. Two-thirds (73.7%) of mothers had a Bachelor's degree or above, while 28.0% of the mothers had a monthly income lower than 4,500 yuan.

**Table 1:** Maternal and child characteristics by the ADBB score.

	**Total**	**ADBB <5**	**ADBB ≥ 5**	***P***
	**(*N* = 114)**	**(*N* = 95)**	**(*N* = 19)**	
**Maternal characteristics**				
Maternal age, mean (SD)	30.2 (4.0)	30.1 (4.0)	31.0 (3.9)	0.336
Maternal education, *n* (%)				0.123^a^
High school or less	10 (8.8)	10 (10.5)	0	
College	20 (17.5)	18 (18.9)	2 (10.5)	
Undergraduate	63 (55.3)	51 (53.7)	12 (63.2)	
Master or above	21 (18.4)	16 (16.8)	5 (26.3)	
Maternal income, *n* (%)				0.599^a^
<1,500 CNY	12 (10.5)	11 (11.6)	1 (5.3)	
1,501–4,500 CNY	20 (17.5)	17 (17.9)	3 (15.8)	
4,501–9,000 CNY	43 (37.7)	34 (35.8)	9 (47.4)	
>9,000 CNY	28 (24.6)	24 (25.3)	4 (21.1)	
Refuse to answer	11 (9.6)	9 (9.5)	2 (10.5)	
Abnormal SAS or SDS, *n* (%)	28 (26.4)	23 (26.4)	5 (26.3)	0.991
Cesarean delivery, *n* (%)	32 (28.1)	29 (30.5)	3 (15.8)	0.089
Gestational age at delivery, *n* (%)				
37–38 W	36 (31.6)	29 (30.5)	7 (36.8)	0.551
39–41 W	78 (68.4)	66 (69.5)	12 (63.2)	
**Child characteristics**				
Child age, *n* (%)				0.013^a^
3 M	22 (19.3)	21 (95.5)	1 (4.5)	
6 M	37 (32.5)	33 (89.2)	4 (10.8)	
12 M	32 (28.1)	24 (75.0)	8 (25.0)	
18–24 M	23 (21.2)	17 (73.9)	6 (26.1)	
Boys, *n* (%)	53 (46.5)	40 (42.1)	13 (68.4)	0.011
Birth order, *n* (%)				
First birth	69 (60.5)	58 (61.1)	11 (57.9)	0.760
Second or later birth	45 (39.5)	37 (38.9)	8 (42.1)	
Birth weight z-score, mean (SD)	0.04 (0.8)	0.06 (0.8)	−0.04 (0.8)	0.607
Difficult temperament, *n* (%)	31 (31.1)	26 (30.6)	5 (35.7)	0.509
Abnormal ASQ:SE score, *n* (%)	12 (10.5)	0	12 (63.2)	<0.001^a^
ASQ-3 delay in each domain, *n* (%)	*N* = 107	*N* = 89	*N* = 18	
Communication	6 (5.6)	5 (5.6)	1 (5.6)	1.000^a^
Gross motor	12 (11.2)	8 (9.0)	4 (22.2)	0.102^a^
Fine motor	15 (14.0)	11 (12.4)	4 (22.2)	0.270^a^
Problem-solving	17 (15.9)	16 (18.0)	1 (5.6)	0.317^a^
Personal-social	14 (12.3)	12 (13.5)	2 (11.1)	1.000^a^
At least one delay	34 (31.8)	27 (30.3)	7 (38.9)	0.419

a *Fisher's exact test was used instead of the χ^2^ test as there were expected frequencies <1 or ≥ 20% of the expected frequencies ≤ 5.*

*ADBB, Alarm Distress Baby Scale; SAS, Self-rating Anxiety Scale; SDS, Self-rating Depression Scale; ASQ:SE, Ages & Stages Questionnaires: Social-Emotional; ASQ-3, Ages & Stages Questionnaires – Third Edition*.

The proportions of children aged 3, 6, 12, and 18–24 months were 19.3, 32.5, 28.1, and 21.2%, respectively. The mean birth weight z-score was 0.04 (SD 0.8), The mean gestational age was 39 weeks (range 37–41, SD 1.1). Overall, 53.5% of the children were female. Comparisons of maternal and child characteristics between the children with an ADBB score ≥ 5 and those with a score <5 are also shown in Table 1. Between these two groups, there was no difference in all characteristics except for child age, gender, and social-emotional development, where higher proportions of older children (*p* = 0.013), boys (*p* = 0.011), and children with an abnormal ASQ:SE score (*p* < 0.001) were observed in the group with an ADBB score ≥ 5. Similar child development, measured by the proportion of having difficult temperament and domain-specific developmental delay, was also observed between these two groups. In addition, no differences in child temperament scores or maternal SAS or SDS scores were found between the children with an ADBB score ≥ 5 and <5 (Supplementary Table 1).

### Distribution of the ADBB Item Score

**Figure 1:**
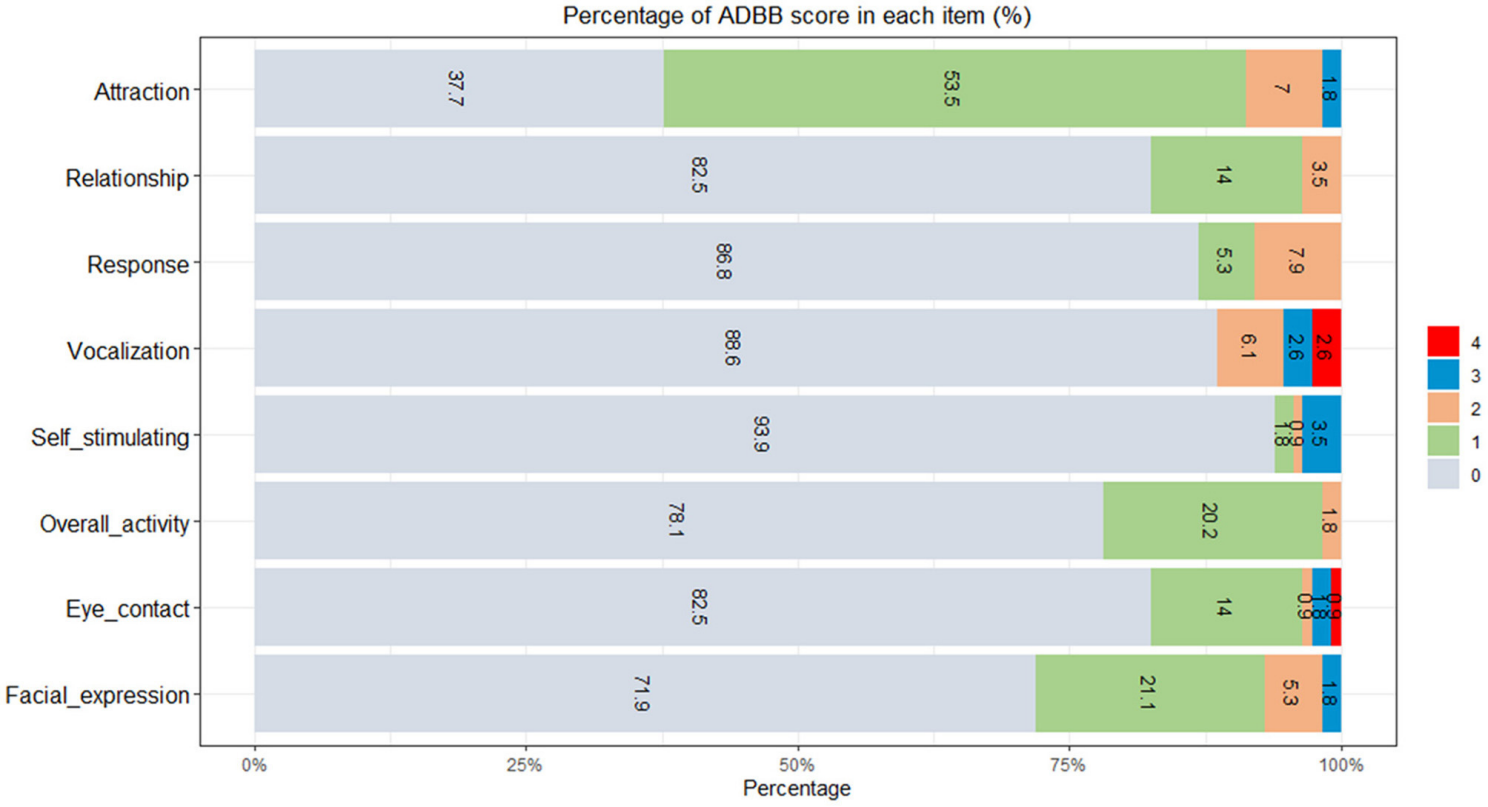
Distribution of the alarm distress baby scale (ADBB) item score (*N* = 114).

The score of each of the eight items ranged from 0 (definite normal behavior) to 4 (definite social withdrawal behavior). On the self-stimulating item, 93.9% (107/114) of the children had a score of 0, while the corresponding proportion for the attraction item was 37.7% (43/114) (Figure 1).

### Distribution of the ADBB Total Score

**Figure 2:**
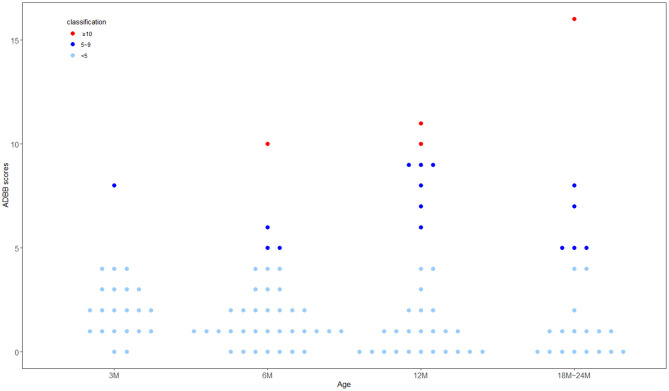
Distribution of the alarm distress baby scale (ADBB) total score by age (*N* = 114).

The mean of the ADBB total score was 2.44, with a standard deviation of 2.91, a median of 1, and a range of 0–16. The distribution of the ADBB total score is shown in Figure 2. About 16.7% (19/114) of the children were considered socially withdrawn, defined as an ADBB score ≥ 5. Among these children, 3.5% (4/114) had severe sustained withdrawal behavior, defined as an ADBB score ≥ 10. The proportion of children with an ADBB score ≥ 5 tended to increase with age.

### Age-Specific Development in Children With an ADBB Score ≥ 5 vs. <5

Table 2 shows the mean score of each ASQ-3 domain and the ASQ:SE in children aged 3, 6, 12, and 18–24 months, respectively, by their ADBB score. Child development measured by the ASQ:SE was poorer in children with an ADBB score ≥ 5 than those with a score <5 across all age groups. Only one in 22 (4.5%) of children aged 3 months had an ADBB score ≥ 5. Compared to peers with a lower ADBB score, this child had better development as measured by the ASQ-3. At 6 and 12 months, the mean ASQ-3 domain score was generally similar between the two groups, except for gross motor at 6 months (27.5 vs. 41.7). At 18–24 months, children with an ADBB score ≥ 5 had generally poorer development, as measured by the ASQ-3, than those with a score <5.

**Table 2:** Distribution of the ASQ-3 (*N* = 107) and ASQ:SE (*N* = 114) domain score [shown as mean (SD)] by age and the ADBB score.

**Parameter**	**ADBB <5**	**ADBB ≥ 5^**a**^**
Age 3 months (*n*, %)	21 (95.5)	1 (4.5)
Communication	44.3 (12.3)	50
Gross motor	41.9 (11.5)	60
Fine motor	32.38 (12.0)	40
Problem-solving	34.29 (11.2)	50
Personal-social	38.1 (10.8)	50
ASQ:SE	28.6 (7.1)	55
Age 6 months (*n*, %)	33 (89.2)	4 (10.8)
Communication	46.9 (7.1)	46.3 (7.5)
Gross motor	41.7 (10.6)	27.5 (11.9)
Fine motor	44.8 (13.3)	43.8 (13.8)
Problem-solving	43.3 (12.9)	36.3 (17.0)
Personal-social	40.3 (11.1)	35 (12.2)
ASQ:SE	27.6 (8.9)	46.3 (11.1)
Age 12 months (*n*, %)	24 (75.0)	8 (25.0)
Communication	46.9 (7.0)	46.3 (7.5)
Gross motor	49.2 (8.7)	48.6 (13.5)
Fine motor	46.6 (10.4)	47.1 (9.1)
Problem-solving	48.9 (14.1)	42.9 (12.9)
Personal-social	42.4 (15.7)	47.9 (9.9)
ASQ:SE	26.5 (9.4)	39.4 (14.7)
Age 18–24 months (*n*, %)	17 (73.9)	6 (26.1)
Communication	47.7 (15.1)	36.7 (19.7)
Gross motor	56.8 (7.7)	44.2 (23.8)
Fine motor	51.2 (12.6)	45.8 (13.9)
Problem-solving	52.7 (7.7)	52.5 (7.6)
Personal-social	54.1 (7.1)	47.5 (8.2)
ASQ:SE	24.2(11.6)	40.8 (14.6)

a *As only one child aged 3 months had an ADBB score ≥ 5, the exact ASQ-3 and ASQ:SE scores of this child are shown.*

*ADBB, Alarm Distress Baby Scale; ASQ-3, Ages & Stages Questionnaires – Third Edition; ASQ:SE, Ages & Stages Questionnaires: Social-Emotional*.

## Discussion

In this pilot study of 114 Chinese children aged 3–24 months, the prevalence of social withdrawal was 16.7%. The prevalence of social withdrawal increased with age, and boys were more likely to be identified as socially withdrawn in our sample. At 18–24 months, children with social withdrawal had generally poorer development (as measured by the ASQ-3) than those without. However, there was no significant difference in the development among younger groups. Specifically, social-emotional development, measured by the ASQ:SE, was poorer in socially withdrawn children across all age groups.

Other studies that investigated the prevalence of social withdrawal in children were mainly based on the European population. About 19% of the children at age 1 year were considered socially withdrawn, with an ADBB score ≥ 5, in the French EDEN cohort (6). Similarly, in this pilot study, we found that the prevalence of social withdrawal was about 17% in young Chinese children. The prevalence of social-emotional development delay, on the other hand, was not consistent across studies. Raman et al. conducted a cross-sectional study of 236 children aged 12–36 months and found that 10% of the sample were considered at risk of social-emotional development delay as measured by the ASQ:SE (21). The Akershus Birth Cohort found that 4.5% of 2-year-old children were considered at risk of social-emotional delay (22). The prevalence of social and emotional problems, measured by the Brief Infant-Toddler Social and Emotional Assessment, was 7.7% in 12-to-36-month-old children in Netherlands (23). In another study in rural China, nearly half (46.2%) of the children were delayed in their social-emotional development (24); this high prevalence might be because many of these children were left-behind children raised by their grandparents in rural areas.

Social withdrawal in young children is likely to be overlooked, and other developmental disorders and emotional problems are difficult to identify. Validated questionnaires can, therefore, improve the ability to identify psychosocial problems of children in community-based pediatric services. It has been reported that other scales, including the ASQ:SE and the Brief Instrument Psychological and Pedagogical Problem Inventory, can also identify toddler's emotional difficulties (25). However, these questionnaires were based only on parents' reports. There are pros and cons for parents to fill out questionnaires. Asking parents to complete the assessment questionnaire can not only improve parents' engagement but also provide the evaluators with a good source of information about their children. On the other hand, however, parents tend to overestimate their children's abilities. The ADBB is designed for healthcare workers to observe and assess social withdrawal behavior in children aged 2–24 months, in the context of routine pediatric examinations or during specific psychological assessments (1). In 2018, Smith-Nielsen et al. found it feasible to increase the use of the ADBB in primary healthcare centers; most (92%) of healthcare workers reported that the scale had made a positive contribution to their work (26). So far, there is no sufficient evidence on the correlation between the ADBB and other socioemotional scales. By comparing the ADBB with the ASQ:SE, our study suggests that the Chinese version of the ADBB may be a good tool to assess social withdrawal behavior in young children.

Children with social withdrawal behavior may have higher risks of impaired motor, language, and social development. Guedeney et al. (6) found that language and motor skills at age 1 year were poorer in children with an ADBB score ≥ 5 than those with a score below 5. It is also suggested that temperament is not only directly related to the child's behavior but also indirectly affects the child's stress response through its influence on the mother's attitude (27). Depression of the caregiver was also found negatively associated with children's social-emotional development (24). Springer et al. showed that 42.2% of the mothers with HIV had depressive symptoms, and one-third (31%) of their children (HIV-exposed but uninfected) had neurodevelopmental delay (28). Higher maternal sensitivity, fewer depressive symptoms, and better infant responsiveness have been found associated with a better mother-child relationship (7, 29). In this study, although not statistically significant, the prevalence of difficult temperament in children with an ADBB score ≥ 5 was 5% higher than that in those with a score below 5.

Social withdrawal in infancy and early childhood shares some common behavioral characteristics with autism spectrum disorder (ASD) and hikikomori (1, 8, 30–32). However, these three conditions appear in different age groups, and thus the influence on cognitive and psychological development may vary (33, 34). Although a high ADBB score might be an early signal for ASD and hikikomori in later life, there is a lack of evidence, especially from prospective studies, that supports an association of social withdrawal in infancy and early childhood with subsequent ASD and hikikomori. Beyond the important clues provided by our study, well-designed population-based prospective studies are needed to reveal such potential associations.

A strength of our study is that the samples were from a large prospective cohort, where information on prenatal and postnatal characteristics was prospectively collected to reduce information bias. In addition, our on-site evaluators met the supervision team for guidance on a regular basis during the period of data collection, which also helped to improve the quality of the data.

Admittedly, certain limitations should be noted in our study. First, as a pilot study, our sample size was relatively small. Considering the compliance of children and parents over the long-time face-to-face survey, convenient sampling was adopted in our study to select an appropriate sample from the routine follow-up of the BIGCS. As the direct calculation of sample size was not applicable in this pilot study, we used the sample size estimation method for factor analysis, which is widely used in studies evaluating scales. For factor analysis, the recommended minimum sample size ranges from 3 to 20 times the number of items (35). As the ADBB has 8 items, the appropriate sample size is recommended to be 50–160. Thus, the current sample size (*N* = 114) was enough for our study. Secondly, our study was conducted in a hospital setting, and some children might have the “white-coat” effect. Therefore, the prevalence of social withdrawal of these children in our study might have been overestimated. Lastly, as the normative ADBB score cut-off is not based on Chinese children, we still used an ADBB score ≥ 5 as the cut-off for identifying social withdrawal in our population, which might have been subject to misclassification. However, as the overarching aim of using the ADBB was to screen out the children at high risk of social withdrawal, reducing the type II error was important for this aim. As the mean (SD) of the ADBB score was 2.44 (2.91) in our sample and 1.9 (2.5) in the normative sample (3), respectively, and the median was 1 in both samples, it is unlikely that the cut-off of 5 used in our study underestimates the prevalence of social withdrawal in our population. We also plan to conduct a validation study with a larger sample size to further evaluate this cut-off in young Chinese children.

In conclusion, this pilot study found that the prevalence of social withdrawal in young Chinese children was largely similar to that reported in the European population. The prevalence of social withdrawal increased with age, and boys were more likely to be identified as socially withdrawn. Children identified as socially withdrawn also tended to have poorer social-emotional development. This pilot study provides preliminary information for the design of a subsequent validation study to further investigate whether the ADBB is a useful instrument for screening social withdrawal in Chinese children.

## Data Availability Statement

The original contributions presented in the study are included in the article/Supplementary Material, further inquiries can be directed to the corresponding author.

## Ethics Statement

The studies involving human participants were reviewed and approved by Institutional Ethics Committee of Guangzhou Women and Children's Medical Center. Written informed consent to participate in this study was provided by the participants' legal guardian/next of kin.

## Author Contributions

XQ designed the study and directed its implementation. AG developed the original version of the scale used in this study. FZ carried out data analysis and drafted the manuscript. PH contributed to the writing of the paper. YG, LF, ML, XL, XW, and ST were involved in study design, questionnaire development, data collection, and follow-up of participants. JL managed the data. SS, AG, and AD revised the manuscript. All authors critically revised the manuscript and approved the final version.

## Conflict of Interest

The authors declare that the research was conducted in the absence of any commercial or financial relationships that could be construed as a potential conflict of interest.

## Abbreviations

ADBB: Alarm Distress Baby Scale
ASQ-3: Ages & Stages Questionnaires, Third Edition
ASQ:SE: The Ages and Stages Questionnaire: Social-Emotional
SPSS: Statistical Package for Social Sciences
SDS: Self-rating depression scales
SAS: Self-rating anxiety scales.
