# Supplementary material for: Prevalence and Characteristics of Social Withdrawal Tendency Among 3–24 Months in China: A Pilot Study
Source: Front Psychiatry. 2021 Jun 16;12:537411. doi: 10.3389/fpsyt.2021.537411 (PMC8242944; doi:10.3389/fpsyt.2021.537411)
Supplement: Supplementary file 1 [file Table_1.DOC]

**Supplementary Table 1** Infant temperament and maternal mood by ADBB scores (shown as mean (SD))

| **Parameter** | **ADBB<5** | **ADBB≥5** | ***P*** |
| --- | --- | --- | --- |
| Temperament | N=89 | N=10 |  |
| Activity level | 3.49 (0.71) | 3.30 (0.77) | 0.589 |
| Rhythmicity | 4.04 (0.62) | 4.15 (0.80) | 0.917 |
| Withdrawal | 3.97 (0.68) | 3.97 (0.75) | 0.921 |
| Adaptability | 4.13 (0.87) | 4.16 (0.71) | 1.000 |
| Intensity of reaction | 3.58 (0.57) | 3.60 (1.01) | 0.140 |
| Quality of mood | 3.90 (0.67) | 3.59 (1.01) | 0.307 |
| Persistent | 3.59 (0.55) | 3.35 (0.59) | 0.272 |
| Distract | 3.80 (0.63) | 3.77 (0.82) | 0.749 |
| Threshold | 3.42 (0.75) | 3.38 (0.62) | 0.991 |
| Maternal mood | N=91 | N=11 |  |
| SAS | 40.52 (7.27) | 44.43 (12.68) | 0.567 |
| SDS | 44.53 (9.88) | 46.25 (11.40) | 0.683 |

SDS: Self-rating depression scales, SAS: Self-rating anxiety scales.
